# WIP, YAP/TAZ and Actin Connections Orchestrate Development and Transformation in the Central Nervous System

**DOI:** 10.3389/fcell.2021.673986

**Published:** 2021-06-14

**Authors:** Inés M. Antón, Francisco Wandosell

**Affiliations:** ^1^Departamento de Biología Molecular y Celular, Centro Nacional de Biotecnología (CNB-CSIC), Madrid, Spain; ^2^Centro de Investigación Biomédica en Red de Enfermedades Neurodegenerativas (CIBERNED), Madrid, Spain; ^3^Departamento de Neuropatología Molecular, Centro de Biología Molecular “Severo Ochoa”, Universidad Autónoma de Madrid – Consejo Superior de Investigaciones Científicas, Madrid, Spain

**Keywords:** glioblastoma, cytoskeleton, neuritogenesis, axonogenesis, nuclear actin, Hippo pathway

## Abstract

YAP (Yes-associated protein) and TAZ (transcriptional coactivator with PDZ-binding motif) are transcription co-regulators that make up the terminal components of the Hippo signaling pathway, which plays a role in organ size control and derived tissue homeostasis through regulation of the proliferation, differentiation and apoptosis of a wide variety of differentiated and stem cells. Hippo/YAP signaling contributes to normal development of the nervous system, as it participates in self-renewal of neural stem cells, proliferation of neural progenitor cells and differentiation, activation and myelination of glial cells. Not surprisingly, alterations in this pathway underlie the development of severe neurological diseases. In glioblastomas, YAP and TAZ levels directly correlate with the amount of the actin-binding molecule WIP (WASP interacting protein), which regulates stemness and invasiveness. In neurons, WIP modulates cytoskeleton dynamics through actin polymerization/depolymerization and acts as a negative regulator of neuritogenesis, dendrite branching and dendritic spine formation. Our working hypothesis is that WIP regulates the YAP/TAZ pools using a Hippo-independent pathway. Thus, in this review we will present some of the data that links WIP, YAP and TAZ, with a focus on their function in cells from the central and peripheral nervous systems. It is hoped that a better understanding of the mechanisms involved in brain and nervous development and the pathologies that arise due to their alteration will reveal novel therapeutic targets for neurologic diseases.

## Introduction

Proper neuritogenesis in post-mitotic neurons is a requisite for dendritic arborization and neuronal function. Neurite extension, sprouting and axonal polarization is directed by microtubule (MT) dynamics and polymerized actin microfilaments (MF), which form actin-rich structures (lamellipodia and filopodia) in the tip of the growth cone. The tip of the growth cone is more a filamentous actin (F-actin) field whereas the intermediate and proximal regions of the growth cone are MT filled (Black and Baas, 1989). These two major cytoskeletal elements have some opposing functions; as a general rule, MT depolymerization prevents or retracts the initial neuritic extension and growth, whereas MF depolymerization leads to generation of multiple axons (Bradke and Dotti, 1999).

In almost all cell types, including neurons, the dynamics of branched MF rely on actin polymerization controlled by the Arp2/3 (actin-related protein) complex and by NPF (nucleation-promoting factors) such as cortactin and N-WASP/WASP (neural/Wiskott-Aldrich syndrome protein) (Padrick and Rosen, 2010). Much less is known about the role of the actin regulator WIP (WASP Interacting Protein) in neuritogenesis, though we recently showed that WIP had unexpected functions in neurons and glia. In the present review we will summarize some of the data linking WIP function with new regulatory aspects of neuritogenesis and its contribution to transformation of astrocytes, another essential component of the nervous system. In addition to the older regulatory functions of WIP in actin polymerization, a few new players have been identified: Yes-associated protein (YAP) and transcriptional coactivator with PDZ-binding motif (TAZ).

### WIP and Actin

WIP was initially described as a regulator of the formation of actin-rich cerebriform projections in B lymphocytes (Ramesh et al., 1997). It is very abundant in hematopoietic cells like lymphocytes and dendritic cells, while lower levels are found in astrocytes, neurons and fibroblasts, where it regulates formation of lamellipodia and filopodia (Antón et al., 2007). WIP binds actin as well as N-WASP and cortactin, regulating their actin-nucleation capability through Arp2/3 (Paunola et al., 2002). Binding of the N-terminal domain of WASP by the WIP C-terminal domain was demonstrated in initial descriptions of the complex (Ramesh et al., 1997), and its contribution to shield WASP proteasome-mediated degradation was identified soon after (Sasahara et al., 2002). Advanced nuclear magnetic resonance structures of purified co-expressed fragments from WASP (a.a. 20–158) and WIP (a.a. 442–492) have confirmed these results and unraveled WIP’s wrapping around WASP (Halle-Bikovski et al., 2018). WIP residues a.a. 454–456 are the major contributor to WASP affinity, while residues a.a. 449–451 have the greatest effect on WASP phosphorylation, and likely its degradation.

WIP binds monomeric globular actin (G-actin) and polymerized F-actin with different affinities (Martinez-Quiles et al., 2001), and their direct interaction depends on the residues ^45^KLKK^48^. In lymphocytes, around 80% of cellular WIP exists in a constitutive complex with WASP, which itself directly binds actin (Koduru et al., 2007). The WIP sequence contains a large number of prolines, which potentiates contact with other proteins including SH3 (src homology 3) domains, which are very common among actin-binding elements. The links between the WIP/WASP complex and the actin cytoskeleton and derived structures are therefore multiple and very relevant. Some of the described WIP-dependent cellular functions such as fibroblast chemotaxis toward PDGF (platelet-derived growth factor) are dependent on WIP’s ability to bind actin, since mutations in the KLKK domain prevent actin co-precipitation, formation of actin-rich dorsal ruffles and impede fibroblast-directed migration (Antón et al., 2003).

We reported WIP expression in the adult mouse brain (cortex, hippocampus and olfactory bulb) and cultured embryonic cortical and hippocampal neurons. We demonstrated that WIP acts as a negative regulator of early neurite emission and branching: development of WIP-deficient cortical neurons from knockout embryos was accelerated, whereas neurite protrusion was retarded by WIP-overexpression. In contrast, WIP-deficient neurons did not have modified axon formation, number or complexity (Franco et al., 2012). The observed phenotype was accompanied by mislocalization of actin NPFs such as N-WASP and cortactin. Thus, our results support that a cooperative action of WIP/N-WASP/cortactin and the Arp2/3 complex are essential for control of actin polymerization, a process required in the initial steps of neuritogenesis for proper neuronal morphogenesis and neuronal network formation. Therefore, we could propose that the growth cone of WIP-deficient neurons may have a more active polymerization dynamic, and this activity could be translated into more robust integrin-associated signaling, locally. This could explain the effect of better initiation of neurite protrusion and axonogenesis of WIP-deficient neurons, although these initial differences with the wild-type neurons, it smoothest out over time, WIP-knockdown dissociated hippocampal neurons maintained both the number of primary neurites and total neuritic length higher than wild-type neurons (Franco et al., 2012).

From these data, several working hypotheses may be proposed about how WIP controls the process of neuritogenesis. A structure-related option based on the regulatory role of actin dynamics points to WIP as a negative regulator of the actin polymerization complex Arp2/3 cortactin-N-WASP. A second option focuses on signaling, with WIP modulating cytoplasmic pathways that control neuritic outgrowth and promote axonal polarity, such as PI3K-Akt-GSK3 pathway, Par3/Par6/aPKC or the multiprotein complex mTORC1 (Jiang and Rao, 2005; Garrido et al., 2007).

One of the main cytoplasmic regulators of actin polymerization is the Rho-GTPase superfamily, which is regulated by the PI3K-Akt pathway, among others. For example, RhoA, Rac1, and Cdc42 are major modulators of the cytoskeleton that act through actin-binding proteins such as N-WASP and WIP (Antón et al., 2020). It has been reported that active Cdc42-GTP interacts with WASP and N-WASP, thereby increasing its nucleation activity (Aspenström et al., 1996; Martinez-Quiles et al., 2001). In neurons, a lack of WIP increased dendritic spine size and filamentous actin content in a RhoA-dependent manner (Franco-Villanueva et al., 2014). Furthermore, stimulated by several growth factors the Akt-mTORC1-S6K pathway is involved in actin cytoskeleton reorganization (Jaworski and Sheng, 2006) and S6K promotes actin filament crosslinking and stabilization by directly binding F-actin, depending on the level of S6K phosphorylation (Moresco et al., 2005). WIP not only plays a cytoskeletal role, but also governs signaling through mTORC1- and Abl-dependent modulation of the S6K pathway, which controls neuritic extension and branching (Franco-Villanueva et al., 2015).

All these data strongly suggest that WIP may play two complementary roles in neurogenesis: regulating actin polymerization levels through N-WASP and by modulating the signaling activity of mTORC1.

### YAP/TAZ and Actin

Actin dynamics regulate axonogenesis and neuronal polarity, as demonstrated by the generation of multi-axons in primary neurons subjected to actin depolymerization, favored either by latrunculin or cytochalasin D (Bradke and Dotti, 1999; Inagaki et al., 2001). Actin dynamics are controlled by well-characterized extracellular signaling pathways including tyrosine kinase receptors, adhesion molecules and neurotransmitter receptors, which use Rho-GTPases as downstream elements in almost all cases. Subsequently, Rho-GTPases exert functions via a plethora of conserved protein effectors, which can be general or quite specific to the particular GTPase (Hall, 2012). More recently, it has been described how extracellular stimuli are mechanotransduced and regulated by a new set of elements: YAP and TAZ (Chan et al., 2005). YAP/TAZ are transcription co-regulatory proteins that respond to physical signals by activating transcription, particularly that of genes involved in extracellular matrix remodeling and cytoskeleton reorganization. YAP/TAZ were initially described as the final effectors of the Hippo pathway (highly conserved from *Drosophila* to mammals), although they can also perform Hippo-independent activities. In contrast to ordinary transduction signaling based on single ligand-receptor interaction, the Hippo pathway integrates multiple upstream stimuli ignited by soluble factors, activation of adhesion molecules and forces driven by the actin cytoskeleton. Therefore, there is a strong cooperative effect among Hippo signaling with cytoskeletal regulation and a combination of extracellular cues, initiating directed migration and mechanotransduction.

### YAP/TAZ Phosphorylation, Quantity, and Subcellular Localization Regulate Their Activity

**FIGURE 1:**
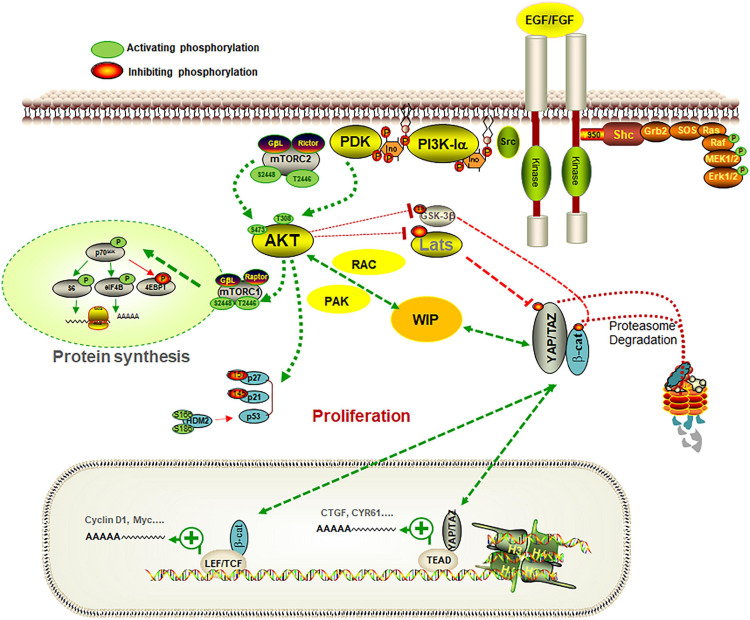
Pathways controlling the glia-glioma transformation. The scheme represents some of the signaling pathways involved in the regulation of the role of WIP in gliomas. WIP drives a mechanism that stimulates growth signals, promoting YAP/TAZ and β-catenin stability in a Hippo-independent fashion, and allows cells to coordinate processes such as proliferation, stemness, and invasiveness, which are key factors in cancer progression. When gliomas were grown in a defined medium containing EGF and FGF as growth factors, PI3K inhibition or Akt knockdown reduced their proliferation. WIP is overexpressed in gliomas and its downregulation decreased YAP/TAZ levels and their targets (such as CTGF or CYR61), in parallel with a reduction in proliferation and stem markers such as nestin. The effect of WIP reduction was compensated, at least in part, by over-expression of Rac or PAK. Some elements of the PI3K-Akt-mTORC1 pathway and the connection of Akt with WIP and YAP/TAZ, are represented in this scheme.

In the Hippo pathway, two conserved kinases regulate the activity of YAP/TAZ, designated as serine/threonine-protein kinases 4 and 3 (STK4/3, usually referred to as MST1/2) (Figure 1). These kinases form heterodimers with Salvador homolog 1 (SAV1), an interaction that is required for MST1/2 to phosphorylate not only SAV1 but also MOB kinase activator 1A protein and the serine/threonine-protein kinase LATS1/2 (Chan et al., 2005). Afterward, when the Hippo pathway is activated, LATS1/2 directly phosphorylates YAP and TAZ at multiple sites, preventing their nuclear localization (Ma et al., 2019). This cytoplasmic retention is mostly regulated by the binding of 14–3–3 protein to phosphorylated YAP/TAZ, resulting in YAP/TAZ inhibition. Live multiphoton microscopy showed that during cellular interphase, endogenously tagged Yki (YAP homolog in *Drosophila*) rapidly fluctuates between the cytosol and the nucleus, where it locates to mitotic chromatin (Manning et al., 2018). The Hippo pathway regulates Yki subcellular distribution by regulating its rate of nuclear import. It is tempting to suggest that Yki/YAP may perform nuclear roles in addition to transcriptional regulation, opening up the search for exclusively nuclear binding partners. Regulation of the amount and half-life of YAP/TAZ relies on the successive phosphorylation by casein kinase 1, which leads to β-TrCP–mediated ubiquitination and proteasomal degradation (Liu et al., 2010; Zhao et al., 2010).

In the cytoplasm, YAP/TAZ have been associated with adhesion molecules such as cadherin, or linked to cytoplasmic actin or actin-binding proteins such as the angiomotin family (AMOT) (Zhao et al., 2011). YAP/TAZ enter the nucleus through mechanical stretching of the nuclear membrane and nuclear pore regulation (Elosegui-Artola et al., 2017). When YAP/TAZ are dephosphorylated and translocate into the nucleus (i.e., when the Hippo pathway is inactive), they can act as transcriptional co-regulators. Neither of these proteins contain DNA-binding domains, and consequently they must interact with direct transcription factors. Although their main binding partners are TEAD family transcription factors, some of which differentially regulate brain cortical development (Mukhtar et al., 2020), YAP/TAZ are capable of forming complexes with other transcription factors such as SMAD, RUNX1/2, p63/p73, or OCT4 (Yu et al., 2015).

YAP/TAZ regulate and are regulated by the actin cytoskeleton. For instance, CYR61 and CTGF, two genes whose transcription is usually induced by the complex YAP/TAZ-TEAD, encode extracellular matrix proteins that work as integrin ligands and are functionally important for cell adhesion to the extracellular matrix (Jedsadayanmata et al., 1999). As a general rule, YAP/TAZ control a set of matrix proteins or cytoskeleton remodeling genes and, in addition, the actin cytoskeleton plays an essential role in regulating YAP/TAZ activity by controlling their cytoplasmic–nuclear shuttling.

A second cytoplasmic element that regulates actin and YAP/TAZ activity is the GTPase RhoA. While the conventional regulation of RhoA–mediated actin polymerization is broadly documented, the detailed mechanism of how RhoA specifically inhibits the Hippo kinase cascade is not completely understood. It is known that ROCK (Rho-associated protein kinase) is partially involved, and that F-actin mediates the effect of Rho on Hippo regulation since the disruption/depolymerization of F-actin by latrunculin or cytochalasin D strongly induces LATS1/2-mediated phosphorylation, preventing the nuclear shuttling of YAP/TAZ (Aragona et al., 2013).

### YAP/TAZ–Actin, Nuclear and/or Cytoplasmic Interactions?

In almost all cell types, actin polymerization is regulated by a variety of G- and F-actin-binding, actin-capping and actin-severing proteins (actin regulatory proteins; ARPs), such as gelsolin, CapZ, profilin, cofilin, thymosin β-4, and formins (Lee and Dominguez, 2010). Although the vast majority of actin is cytoplasmic, there is also a nuclear actin component with numerous ARPs (Treisman, 2013) that has long been understudied. Considering all these actin-binding proteins, it is important to remember that YAP/TAZ transcription is regulated by F-actin-capping and -severing proteins such as capZ, cofilin, and gelsolin. Accordingly, knockdown of any of these proteins increases the expression of YAP/TAZ target genes, such as CYR61 or CTGF (Aragona et al., 2013). In many cell types, nuclear actin primarily acts in complex with other proteins of the ARP family, such as the Arp2/3 complex (Goley et al., 2006; Yoo et al., 2007), formins (Parisis et al., 2017), or N-WASP, which participates not only as a structural/cytoskeletal element but also regulates RNA polymerase II-dependent transcription (Wu et al., 2006).

Interestingly, the pool of nuclear F-actin may exert a role in cell cycle regulation through control of chromatin organization at mitotic exit (Baarlink et al., 2017), and it may combine its regulatory effect with the contribution of branched actin networks from the cell cortex to control cell cycle progression in mammary epithelial cells (Molinie et al., 2019). Therefore, considering that numerous ARPs controlling actin dynamics are present both in the cytoplasm and nucleus, some questions about the exact role of nuclear actin in the regulatory activity of YAP/TAZ have not been fully addressed. It is well established that YAP/TAZ nuclear transit is mediated by the actin polymerization stage, however cytochalasin D and latrunculin treatment can simultaneously affect cytoplasmic and nuclear actin. Thus, the question about whether actin is only necessary for the nuclear shuttling of YAP/TAZ, or is playing an additional role in the transcriptional activity of YAP/TAZ is still an open question (Piccolo et al., 2014).

It appears that YAP/TAZ regulation is not directed by the total levels of F-actin, but rather by its subcellular organization, fine structure, tension and resistance offered by the cytoskeleton and by the whole nucleus. This dual cytoplasmic/nuclear role is not exclusive to YAP/TAZ, as other similar co-transcriptional regulators have been described, for instance β-catenin or myocardin-related transcription factor (MRTF), through the TCF family of transcription factors and the transcription mediator serum response factor (SRF), respectively (Olson and Nordheim, 2010; Nusse and Clevers, 2017).

The MRTF and YAP transcriptional pathways contribute to the response to cell proliferation and mechanotransduction. Several observations suggest that MRTFs and YAP/TAZ may functionally interact despite not sharing a common DNA targeting factor. In cancer-associated fibroblasts (CAFs), the MRTF–SRF and YAP pathways are required for the contractile and pro-invasive properties of these cells. It has been reported that in CAFs, expression of direct MRTF–SRF genomic targets is also dependent on YAP–TEAD activity and, conversely, YAP–TEAD target gene expression depends on MRTF–SRF signaling. In normal fibroblasts, expression of activated MRTF versions induces YAP, while activated YAP stimulates MRTF. Cross-talk between the pathways requires recruitment of MRTF and YAP to DNA via their respective DNA-binding partners (SRF and TEAD), and is therefore indirect. However, YAP/TAZ mechanotransduction differs substantially from actin regulation of the MRTF family, as a sensor of F-actin/G-actin ratio binds directly to free nuclear G-actin in a manner that inhibits its association with SRF (Foster et al., 2017).

It has also been reported that WIP can promote nuclear transit of MRTF-SRF via actin polymerization (Ramesh et al., 2014), similar to YAP/TAZ. As both transcription factors depend on actin polymerization to reach the nucleus and WIP contributes to modify the ratio G/F-actin, we considered the possibility that WIP regulates the subcellular distribution of YAP/TAZ through an indirect mechanism based on the levels of cellular F-actin. In contrast, the interaction β-catenin/TCF with YAP/TAZ is direct; some initial data indicate that the Hippo pathway genetically and functionally interacts with Wnt/β-catenin signaling (Varelas et al., 2010). Reports demonstrated a novel mechanism through which Hippo signaling inhibits Wnt/β-catenin input; YAP/TAZ binds to β-catenin, thereby suppressing Wnt-target gene expression. YAP phosphorylation stimulated by the activated Hippo pathway induces cytoplasmic retention of YAP, which is required for the YAP-mediated inhibition of Wnt/β-catenin signaling (Imajo et al., 2012). More recently, an “alternative Wnt-YAP/TAZ signaling axis” was based on Wnt5a/b and Wnt3a, which are potent activators of the critical mediators YAP/TAZ (Park et al., 2015). Moreover, another report indicated that YAP could directly interact with β-catenin in the nucleus, thus forming a transcriptional YAP/β-catenin/TCF4 complex. This transcriptional complex was confirmed by target genes of this complex Lgr5 and cyclin D1 (Deng et al., 2018).

Several data support common regulatory steps between YAP/TAZ and β-catenin. When the Wnt pathway is inactive, YAP/TAZ are sequestered in the β-catenin destruction complex where they recruit βTrCP, which is needed for β-catenin inactivation. In contrast, when Wnt is activated, YAP/TAZ are released from the complex and accumulate in the nucleus (Azzolin et al., 2014).

### YAP/TAZ and Neuronal Development

The extracellular matrix protein/integrin interaction serves as a first responder to collect mechanosignals and transmit the information to the regulatory machinery of YAP/TAZ, possibly not only through the Hippo pathway (Hu J. K. H. et al., 2017). It is tantalizing to propose that YAP/TAZ may play a role in the regulation of axonogenesis induced by the extracellular matrix. However, the exact mechanism underlying how YAP/TAZ are regulated by various mechanical/extracellular signals in neurogenesis is far from known.

Current data indicate an important role of YAP/TAZ in neuronal development. Development of the nervous system is based on proliferation of neural stem cells (NSCs) and posterior differentiation into diverse neural lineages. NSCs are the group of self-renewing cells that can generate neurons and several glial cell types during embryonic development (Beattie and Hippenmeyer, 2017). Recently, several studies have suggested an essential role of the Hippo signaling pathway in regulation of NSC proliferation. For instance, downregulated Hippo signaling ensured translocation of YAP inside the nucleus, thereby transcribing genes associated with cellular proliferation. Prolonged YAP/TAZ activation also enhances the stemness (Panciera et al., 2016) and delays differentiation of NSCs. In a mouse model and a murine primary neuronal culture system, it has been reported that overexpression of YAP/TAZ promotes NSC characteristics *in vivo* in the stem cell niche, and increases the size of cultured neurospheres. Moreover, this YAP/TAZ function restricting differentiation is dependent on the transcriptional co-activator TEAD (Han et al., 2015; Robledinos-Antón et al., 2020).

**FIGURE 2:**
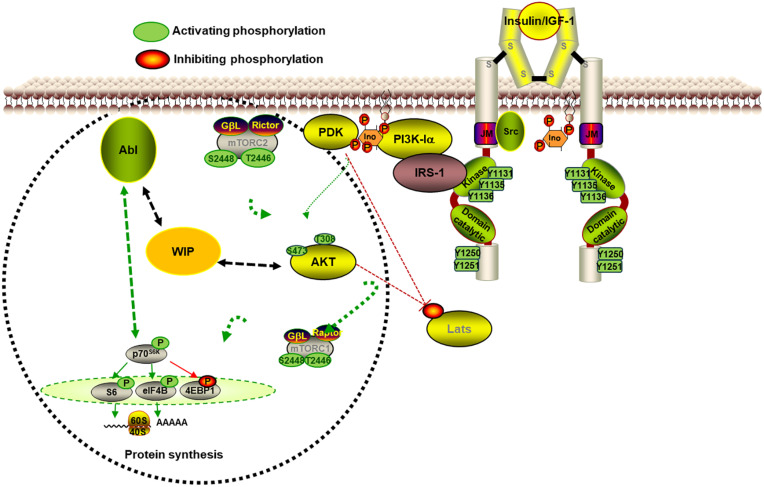
WIP controls neurite initiation and extension in primary neurons. The scheme represents some of the signaling involved in the contribution of WIP in neurite extension. The WIP knockdown increased neurite protrusion and elongation in a neuronal model in which the high level of insulin is the main growth factor. WIP not only has a cytoskeleton dependent role, but also governs signaling through mTORC1- and Abl family kinases-dependent modulation of the S6K pathway, which controls neuritic extension and branching.

Considering the previously described interaction between YAP/TAZ and β-catenin and the role of the Wnt pathway in neuronal development, it is tempting to speculate that some of the neuronal actions of Wnt signaling might be a combination of YAP/TAZ and β-catenin-dependent effects. Besides Hippo, pathways including Notch, Shh, FGFs, TGF-β, retinoic acid, and reelin are well-known to regulate NSC proliferation, neurogenesis and gliogenesis. Consequently, the crosstalk between Hippo and some of these signaling pathways and the final impact of these signals on target genes is an open question (Paridaen and Huttner, 2014; Mukhtar and Taylor, 2018; Figure 2).

YAP/TAZ not only conditioned the cell fate of the NSC throughout several signaling pathways, but also reprogrammed the energy metabolism. For instance it has been reported that the Hippo pathway can also affect nucleotide biosynthesis and lipid metabolism through the regulation of *de novo* purine/pyrimidine biosynthesis, gluconeogenesis, amino acid uptake, and cholesterol and lipid biosynthesis (Hu Y. et al., 2017; Santinon et al., 2018).

All the gathered knowledge underlines the need for future studies to clarify how any of these metabolic pathways and their integration with signaling and mechanotransduction cues exert an impact on neuritogenesis, axonal elongation and neuronal maturation.

### Neuritogenesis and NSCs, WIP-YAP/TAZ and Actin

In almost all mammalian cells analyzed, actin forms complexes with other proteins like cofilin or profilin, and they can actively be transported into and out of the nucleus by some members of the importin β superfamily and modulated by Ran GTP (Dopie et al., 2012). Also, as previously mentioned, Arp2/3 and WASP are present in the nucleus where they regulate RNA polymerase II-dependent transcription. In fibroblasts, WIP may localize in the nucleus, and its co-expression with N-WASP caused redistribution of N-WASP, reducing its main nuclear expression and leading to co-localization with WIP in the perinuclear areas (Vetterkind et al., 2002). Whether WIP has a similar effect on WASP subcellular distribution in hematopoietic cells is a relevant question that remains unsolved.

Recently we demonstrated that WIP drives a new mechanism that stimulates growth signals in tumor cells, promoting YAP/TAZ stability that allows cells to coordinate key activities in cancer progression, such as proliferation, stemness and invasiveness. This protein stabilization appears to be Hippo-independent (a LATS mutant did not modify it) and actin-independent (it was not modified by either actin depolymerization or polymerization agents) (Gargini et al., 2016). The specific cellular machinery that determines how actin controls YAP/TAZ activity and whether the cellular effects of WIP expression are solely due to the consequent increase in YAP/TAZ remain open questions. The nuclear transit of MRTF-SRF promoted by WIP is regulated by actin polymerization (Ramesh et al., 2014). However, the question of whether WIP might locally regulate YAP/TAZ transcriptional activity in the nucleus is far from resolved.

In contrast to the pro-oncogenic version of WIP-YAP/TAZ in glioma, WASP and WIP act as onco-suppressor proteins in ALK-dependent lymphomas (Menotti et al., 2019). ALK transforming activity leads to down-regulation of WASP and WIP through transcriptional repression, mediated by STAT3 and C/EBP-b. Interestingly, YAP’s expression and tumoral effects run in parallel to those of WIP: YAP is markedly downregulated in hematological malignancies, including lymphomas, leukemias and multiple myeloma, and is upregulated in cell lines from solid tumors of epithelial origin (Cottini et al., 2014).

As previously indicated, prolonged activation of YAP/TAZ enhances stemness (Han et al., 2015; Panciera et al., 2016). However, the expression of YAP/TAZ in the postnatal brain opens several questions about whether the YAP/TAZ–TEAD complex plays a role beyond maintaining NSC phenotypes, and whether in this context WIP/N-WASP affect the biological/physiological activity of YAP/TAZ, since only limited reports on WIP’s contribution to neuronal development are available. A low number of babies and children suffering a severe immunodeficiency similar to Wiskott-Aldrich syndrome due to mutations in the *WIPF1* gene have been identified (Lanzi et al., 2012; Mansour et al., 2020). Studies related to these patients focused on their immunological alterations and the corresponding therapies, but they did not address the potential neurological consequences derived from mutant WIP malfunction. Interestingly, the *WIPF1* gene was the only common genomic region shared by 6–8 patients suffering from neurological diseases (Mitter et al., 2010). WIP-KO mice provide a useful and relevant model to analyze the potential contribution of WIP to cortical or hippocampal development, as well as its participation in the function of the olfactory bulb, since these three murine brain regions have been shown to express the protein (Franco et al., 2012). Mouse behavioral tests may also shed light on the role of WIP in the reduced reproductive success observed in WIP-KO animals.

Despite a limited understanding of WIP’s contribution to the functionality of the nervous system, the role of WIP-YAP/TAZ in the brain is still an open question. A potentially relevant player is the phosphatase PP2AC, suggested to support cortical neuronal growth and cognitive function by modulating the Hippo-p73 signaling cascade and the glutamate/glutamine cycle (Liu et al., 2018). PP2AC may connect the new WIP-YAP/TAZ pathway, which is heavily activated in some p53 mutant tumor cells and modulated by integrins and Akt signaling (Escoll et al., 2017). Although the putative role of YAP/TAZ in the brain under stressful or disease conditions has barely been studied, it is an important issue to explore, considering the role of the p53 family of proteins in several pathologies. For instance, despite its recognition as a cytoplasmic activator of Arp2/3, WASP has been shown to colocalize in the nucleus with the damage marker γH2AX after DNA-chemical insult (Schrank et al., 2018). Whether WIP also cooperates with WASP in this activity is an attractive research question to pursue.

## Future Perspectives

Neuronal functional morphogenesis relies on coordinated modulation of the reorganization of the actin cytoskeleton by actin-binding proteins like N-WASP and WIP, with the activity of transcriptional co-regulators such as YAP and TAZ, whose nuclear distribution depends on actin. The multiple intertwined connections between these players provide a fascinating working niche with many relevant questions that need to be addressed. For example, is the predominant role of WIP in actin dynamics in complete cellular systems universal, or does it depend on the cell type? Is phosphorylation status of YAP/TAZ the master regulator of their subcellular distribution in mature neurons, astrocytes or glioblastomas? Is the WIP-mediated control of YAP/TAZ stability stronger than the actin-dependent modulation of YAP/TAZ activity? Does WIP indirectly contribute to YAP/TAZ nuclear distribution through actin depolymerization? How is actin and YAP/TAZ crosstalk regulated in a two-way manner? Future work in the field will provide clear answers and, hopefully, lead to novel diagnostic and therapeutic tools for neurological diseases or nervous system tumors.

## Author Contributions

IA and FW: conceived the manuscript, writing of the manuscript, approved its final content, conceptualization, and writing—review and editing. FW: writing original draft preparation. Both authors contributed to the article and approved the submitted version.

## Conflict of Interest

The authors declare that the research was conducted in the absence of any commercial or financial relationships that could be construed as a potential conflict of interest.
